# Correction: Flexible composite film of aligned polyaniline grown on the surface of magnetic barium titanate/polyvinylidene fluoride for exceptional microwave absorption performance

**DOI:** 10.1039/c8ra90064g

**Published:** 2018-07-30

**Authors:** Lujun Yu, Yaofeng Zhu, Yaqin Fu

**Affiliations:** Key Laboratory of Advanced Textile Materials, Manufacturing Technology Ministry of Education, Zhejiang Sci-Tech University No. 928 Second Avenue XiaSha Higher Education Zone Hangzhou 310018 P. R. China yfzhu@zstu.edu.cn +86 571 86843607 +86 571 86843607

## Abstract

Correction for ‘Flexible composite film of aligned polyaniline grown on the surface of magnetic barium titanate/polyvinylidene fluoride for exceptional microwave absorption performance’ by Lujun Yu *et al.*, *RSC Adv.*, 2017, **7**, 36473–36481.

On page 36478 for eqn (5) the final minus sign should be replaced by a plus sign as shown below:5
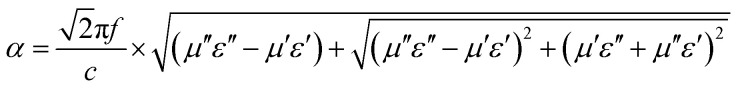


According to this error, Fig. 7a and the associated description were wrong. The correct Fig. 7a is as shown below.

**Fig. 7:**
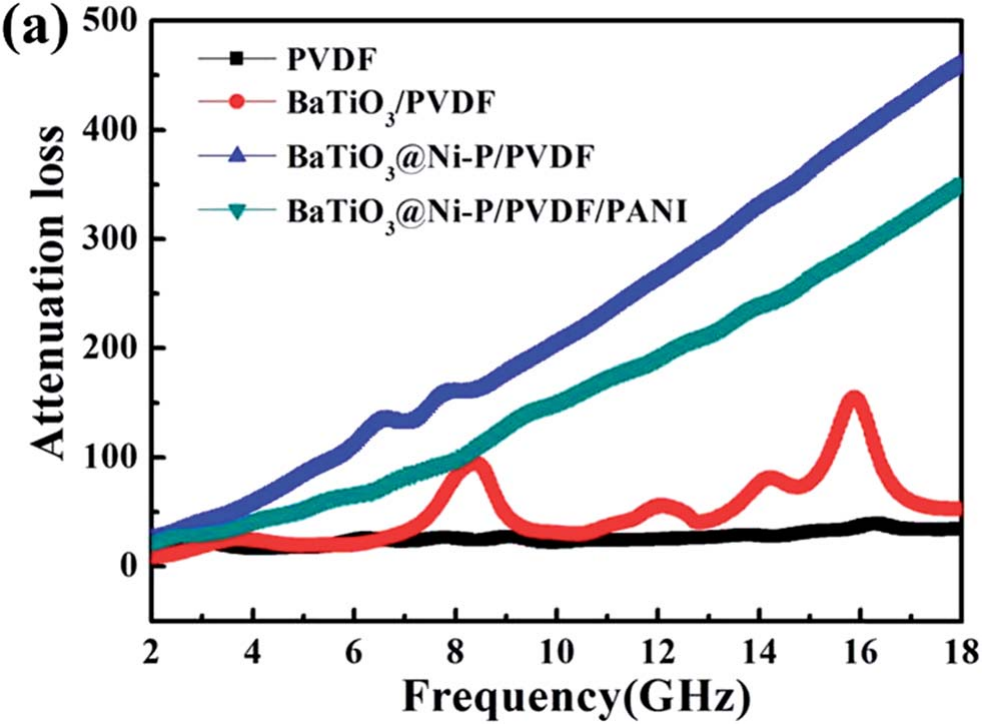
(a) Attenuation constant *α* of the samples.

In addition, the corresponding description in the text (pp. 36478–36479) should be corrected from “the attenuation constant *α* value of BaTiO_3_@Ni–P/PVDF has increased distinctly within the frequency ranges of 2–11.3, 12.7–15.3, and 16.6–18 GHz compared with that of BaTiO_3_/PVDF. In addition, the BaTiO_3_@Ni–P/PVDF/PANI sample clearly exhibits a higher attention constant *α* than that of BaTiO_3_@Ni–P/PVDF within the frequency range of 8.8–18 GHz” to “the attention constant *α* is in the order: BaTiO_3_@Ni–P/PVDF > BaTiO_3_@Ni–P/PVDF/PANI > BaTiO_3_/PVDF > PVDF”.

The Royal Society of Chemistry apologises for these errors and any consequent inconvenience to authors and readers.

## Supplementary Material

